# Pseudocellulitis in oncology patients: A single institutional retrospective analysis investigating the clinical presentation, therapeutic response, and implications for cancer therapy

**DOI:** 10.1016/j.jdin.2023.04.016

**Published:** 2023-06-24

**Authors:** Jolanta J. Pach, Caroline Nelson, Jonathan S. Leventhal

**Affiliations:** Department of Dermatology, Yale School of Medicine, New Haven, Connecticut

**Keywords:** cellulitis, dermatology, lipodermatosclerosis, oncodermatology, oncology, pseudocellulitis

*To the Editor:* Cellulitis is a common clinical diagnosis and the most common dermatologic reason for hospitalization.^1^ However, up to one third of patients diagnosed with cellulitis instead have one of the localized, cutaneous, inflammatory conditions encompassed under the term “pseudocellulitis.”^2^^,^^3^ Pseudocellulitis often presents similarly to cellulitis with erythema, edema, and tenderness but, unlike cellulitis, typically lacks fever and leukocytosis. Examples include venous stasis dermatitis, lymphedema, and contact dermatitis.^2^ Pseudocellulitis may be associated with oncologic therapies, such as acute lipodermatosclerosis and radiation recall.^4^ Distinguishing cellulitis from pseudocellulitis, while challenging, is important to minimize antibiotic usage, hospitalizations, health care spending,^2^ and interruptions of cancer therapy for oncologic patients.

We performed a retrospective chart review of the Yale New Haven Health electronic medical record from 2016 to 2022 to identify all oncologic patients diagnosed with pseudocellulitis by the outpatient oncodermatology or inpatient dermatology consultative services. We sought to characterize the associated oncologic therapy, clinical diagnosis, dermatologic management, and therapeutic response. Outpatient oncodermatology notes were searched by the joint data analytics team using the keyword “pseudocellulitis” and other spelling derivations, and the inpatient consult log was manually searched for these keywords.

Thirty-one patients met inclusion criteria (Table I). Gastrointestinal cancer was the most common underlying malignancy in this cohort (35.5%). Implicated classes of oncologic therapy included cytotoxic chemotherapy (74.2%), targeted therapy (12.9%), combined cytotoxic and targeted therapy (3.2%), radiation therapy (6.5%), and immunotherapy (3.2%). Acute lipodermatosclerosis was the most common condition presenting as pseudocellulitis (74.2%), associated with gemcitabine in 73.9% of cases (Fig 1). Other dermatologic diagnoses of pseudocellulitis included venous stasis dermatitis, injection site reaction/extravasation injury, edema bullae, acute inflammatory edema, radiation recall, acute radiation dermatitis, and irritant contact dermatitis (some patients fit multiple diagnosis categories). Most cases presented within 1 year of initiation of oncologic therapy (87.1%).

**Table I:** Pseudocellulitis in oncologic patients: Demographics, associated oncologic therapy, clinical diagnosis, management, and therapeutic response

Characteristic	Total (*n* = 31)
Mean age (years)	69.5
Sex	
Male	18 (58.1%)
Female	13 (41.9%)
Race∗	
White/Caucasian	25 (80.6%)
Black/African American	4 (12.9%)
American Indian or Alaska Native	1 (3.2%)
Ethnicity∗	
Non-Hispanic	30 (96.8%)
Cancer treatment	
Cytotoxic chemotherapy	23 (74.2%)
Gemcitabine	17 (54.8%)
Pemetrexed	4 (12.9%)
Paclitaxel	1 (3.2%)
Carboplatin and etoposide	1 (3.2%)
Targeted	4 (12.9%)
Rituximab	1 (3.2%)
Lorlatinib	1 (3.2%)
Bortezomib	1 (3.2%)
Enfortumab	1 (3.2%)
Cytotoxic chemotherapy and targeted combined	1 (3.2%)
Eribulin and trastuzumab	1 (3.2%)
Radiation	2 (6.5%)
Immunotherapy	1 (3.2%)
Atezolizumab	1 (3.2%)
Pseudocellulitis diagnosis†	
Lipodermatosclerosis	23 (74.2%)
Venous stasis dermatitis	4 (12.9%)
Injection site reaction/extravasation injury	3 (9.7%)
Edema bullae	2 (6.5%)
Acute inflammatory edema	1 (3.2%)
Radiation recall	1 (3.2%)
Acute radiation dermatitis	1 (3.2%)
Irritant contact dermatitis	1 (3.2%)
Systemic antibiotic use prior to dermatology consultation	24 (77.4%)
Oral	20 (64.5%)
Intravenous	10 (32.3%)
Interruption of cancer therapy	10 (32.3%)
Response to dermatologic therapy	
Improvement	29 (93.5%)
Stable disease	1 (3.2%)
Unknown	1 (3.2%)
Time to improvement	
<1 mo	24 (77.4%)
1-3 mo	5 (16.1%)

∗ One patient had unknown race/ethnicity.

† Some patients fit multiple diagnosis categories.

**Fig 1:**
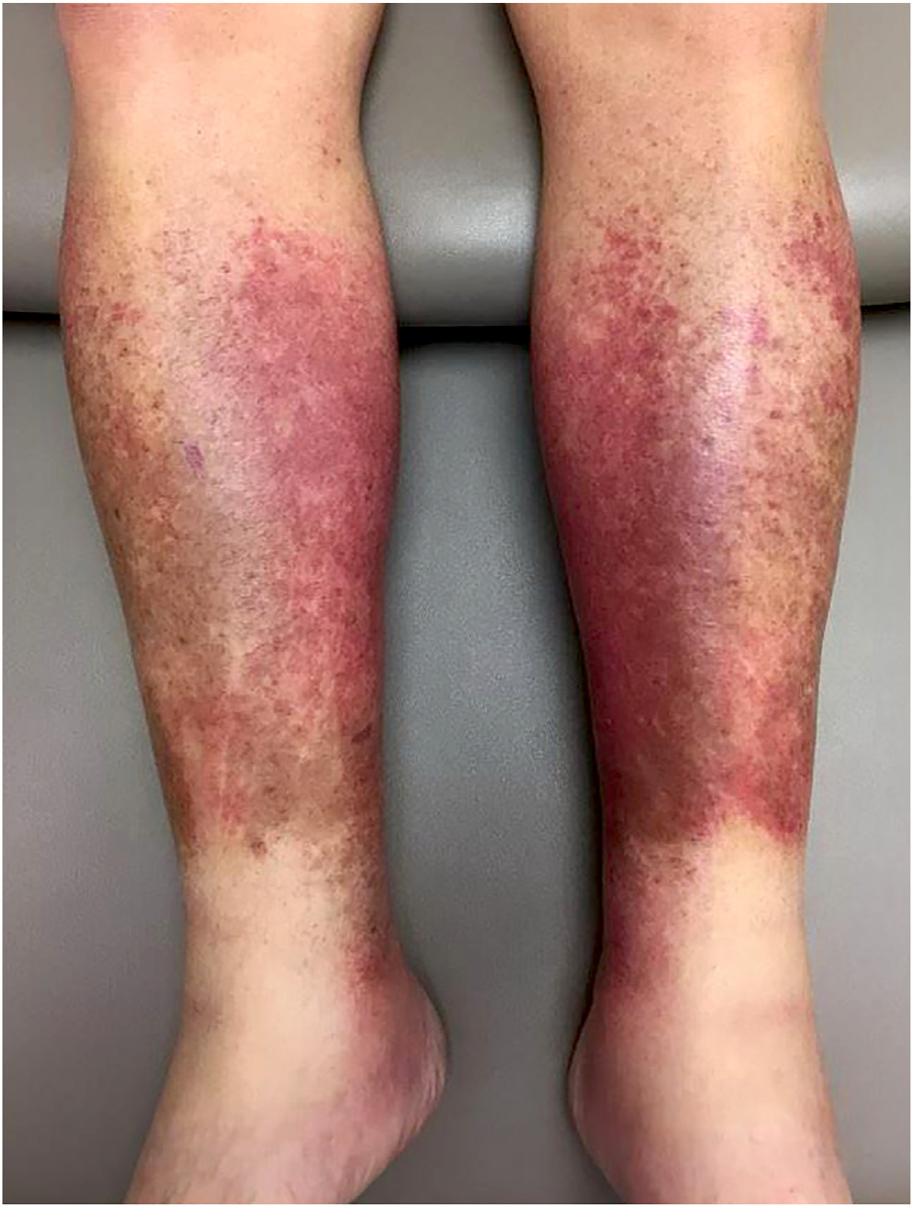
Representative Image of Pseudocellulitis: Gemcitabine-associated acute lipodermatosclerosis.

77.4% of patients received systemic (oral [64.5%] and/or intravenous [32.3%]) antibiotics before referral to dermatology. 32.3% of patients had their cancer therapy held by the oncology or medicine services due to concern for cellulitis. Dermatologic management consisted primarily of topical corticosteroids (87.1% of cases) and resulted in clinical improvement within 3 months (93.5% of cases).

Limitations of our study include its retrospective nature, data from a single institution, and small sample size. Moreover, cases of pseudocellulitis not referred to dermatology or those for which the term “pseudocellulitis” was not used in medical notes were not captured. Upon dermatologic consultation, pseudocellulitis was treated with topical anti-inflammatory agents rather than antibiotics which were frequently prescribed by the referring clinicians, underscoring the challenge in distinguishing it from true cellulitis and the decision to empirically treat with antimicrobial agents in a high-risk population. One prospective study found that early dermatologic consultation for patients with a presumed diagnosis of cellulitis led to lower rates of both antibiotic use and hospitalization.^5^ Our study also highlights the importance of recognizing and managing pseudocellulitis in cancer patients receiving oncologic therapy and underscores the utility of multi-disciplinary oncologic care.

## Conflicts of interest

The authors have no relevant conflicts of interest to report. Dr Leventhal serves on the advisory boards of La Roche-Posay and Sanofi and Regeneron Pharmaceuticals and receives clinical trial funding from Azitra, Inc and OnQuality. Dr Nelson received an unrelated grant from Boehringer Ingelheim.
